# Supplementary material for: Isolation and Characterization of a Novel Hydrophobin, Sa-HFB1, with Antifungal Activity from an Alkaliphilic Fungus, Sodiomyces alkalinus
Source: J Fungi (Basel). 2022 Jun 23;8(7):659. doi: 10.3390/jof8070659 (PMC9322931; doi:10.3390/jof8070659)
Supplement: Supplementary file 1 [file jof-08-00659-s001.zip › jof-1732879-supplementary.pdf]

Isolation and Characterization of a Novel Hydrophobin, Sa-HFB1, With Antifungal Activity From an Alkaliphilic Fungus, *Sodiomyces alkalinus*

Anastasia E. Kuvarina[1], Eugene A. Rogozhin[1,2], Maxim A. Sykonnikov[1], Alla V. Timofeeva[3], Marina V. Serebryakova[3], Natalia V. Fedorova[3], Lyudmila Y. Kokaeva[4], Tatiana A. Efimenko[1], Marina L. Georgieva[1,4] and Vera S. Sadykova[1*]

Supplementary

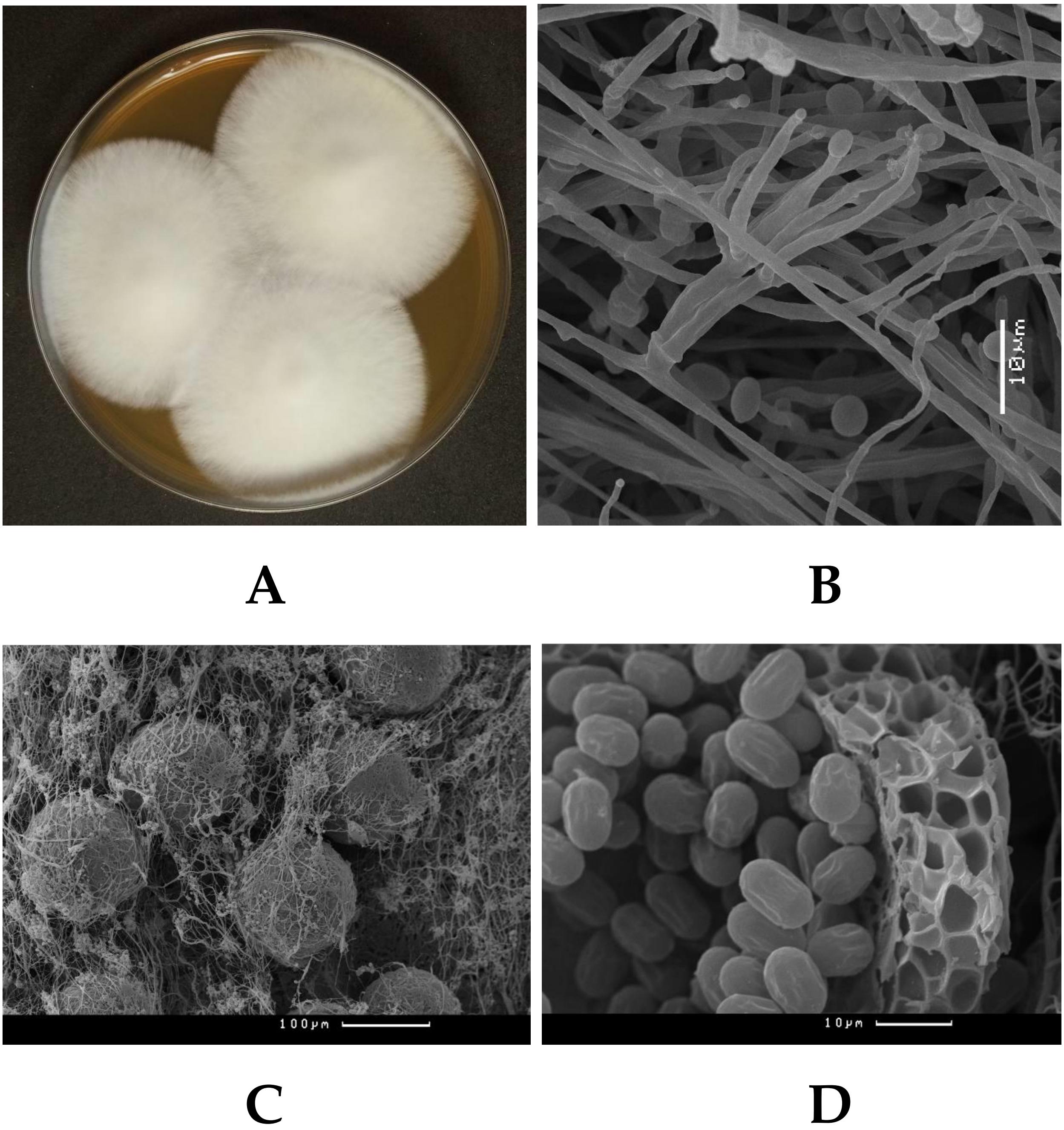

Figure S1. *Sodiomyces alkalinus* Grum-Grzhimaylo, Debets & Bilanenko: A – growth on the alkaline agar medium; B – D scanning electron microscopy: B - conidiophores with 3-5 branches and conidia; C -

cliestothecial ascomata, creeping vegetative hyphae and conidiophores with slimy mass of abundant conidia; D - ascospores and myltilayered peridium of ascomata.

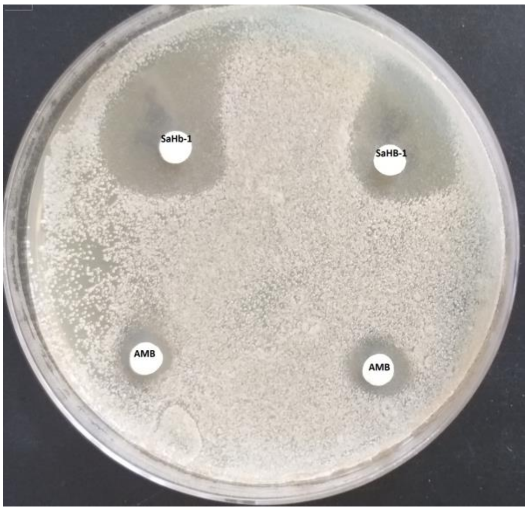

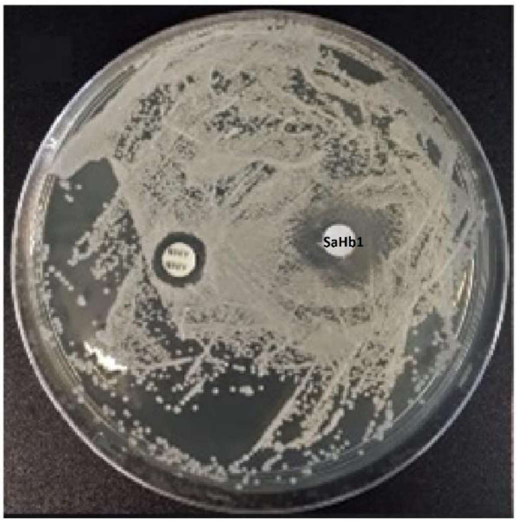

A **B**

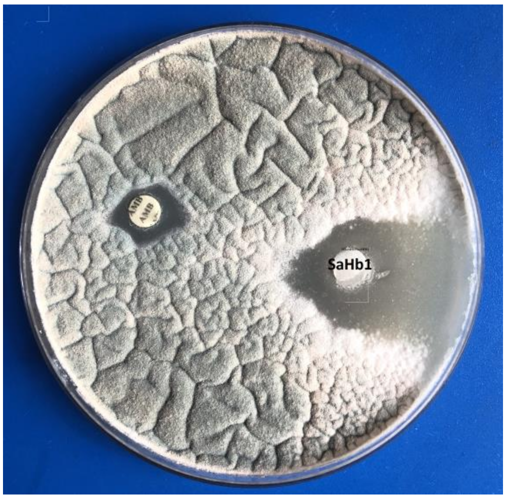

C

Figure S2. Activity of Sa-HFB1 against pathogenic clinical isolates of *Cryptococcus neoformans* 297m (A) *Candida albicans* 1582m (B) and *Aspergillus fumigatus*.

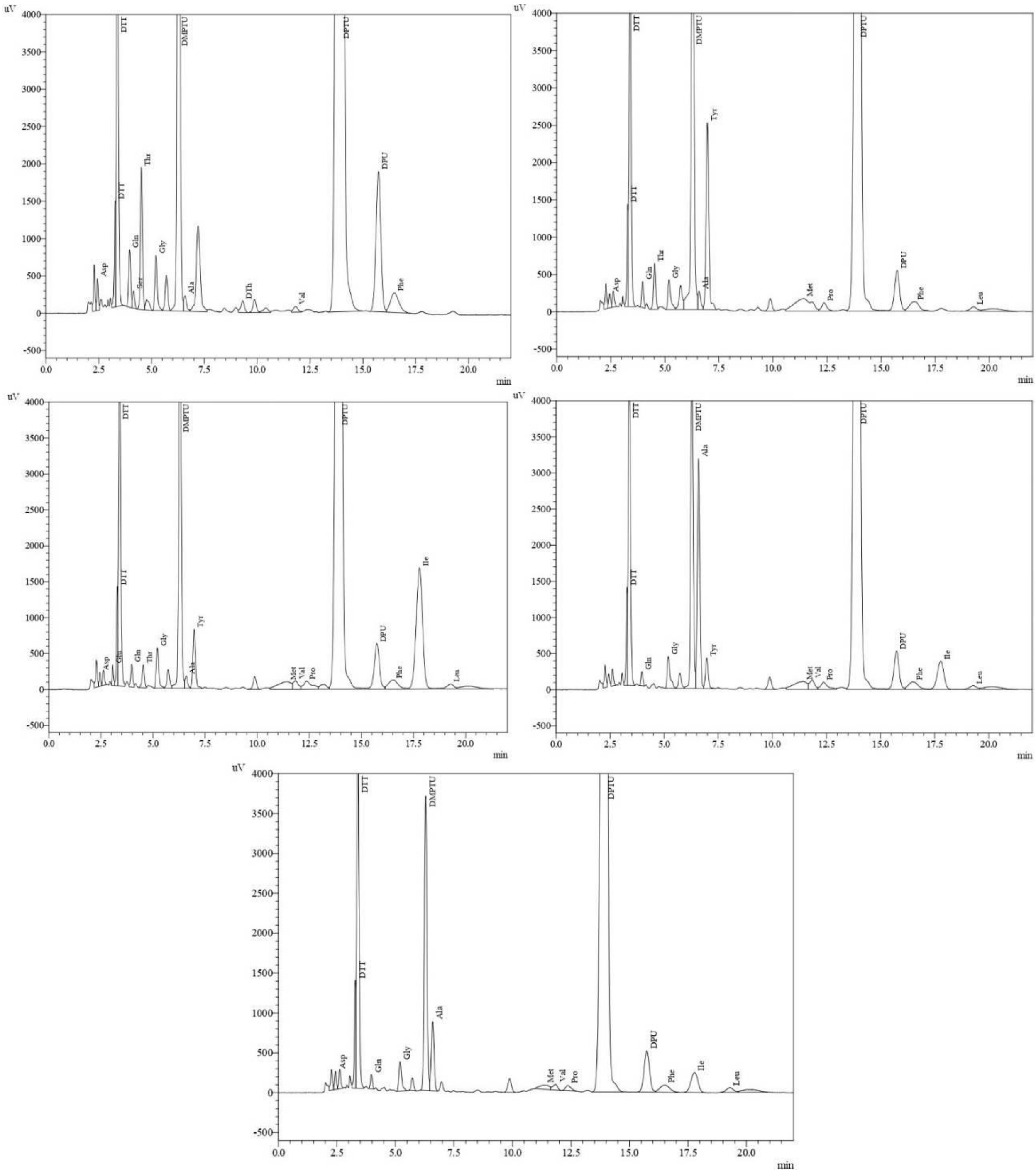

uV
min
DTT
DMPTU
DPTU
DPU
Asp
Gln
Ser
Thr
Gly
Ala
DTh
Val
Phe
Tyr
Met
Pro
Leu
Ile

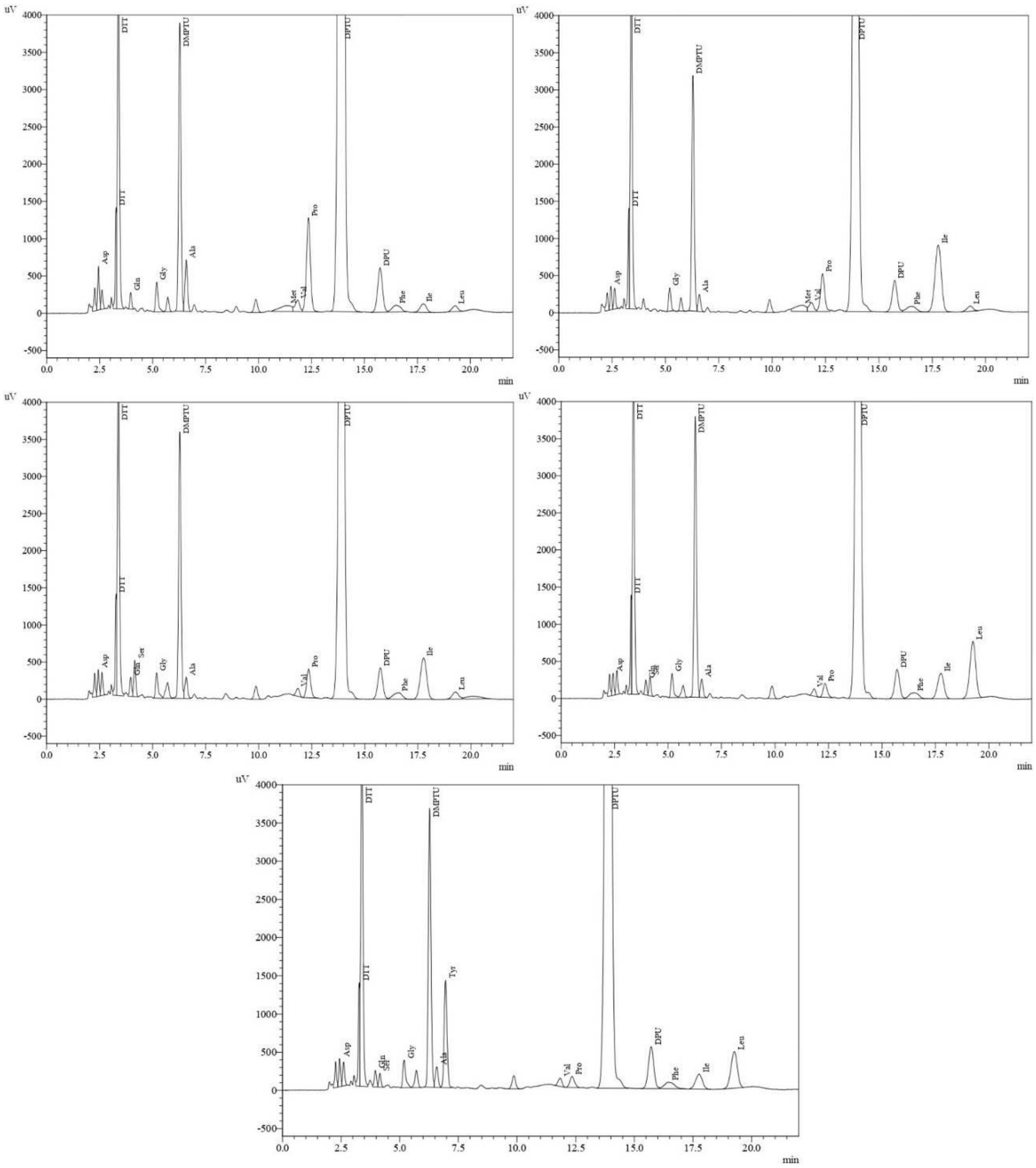
uV
4000
3500
3000
2500
2000
1500
1000
500
0
-500
0.0
2.5
5.0
7.5
10.0
12.5
15.0
17.5
20.0
min
DTT
DMPTU
DPTU
Asp
Gln
Ser
Gly
Ala
Tyr
Met
Val
Pro
DPU
Phe
Ile
Leu

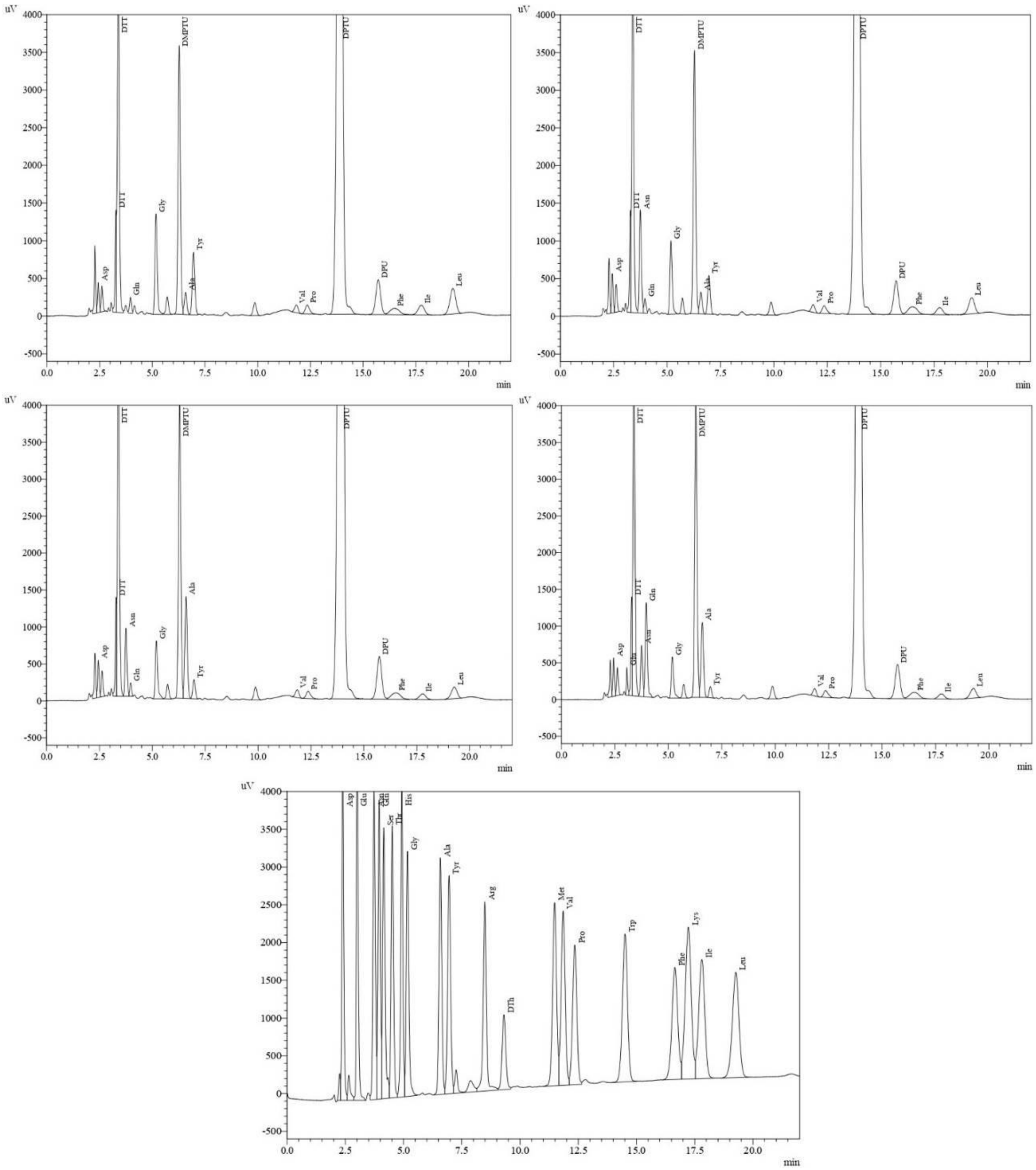

Figure S3. Chromatograms of PTH derivatives of amino acids in N-terminal automated sequencing.

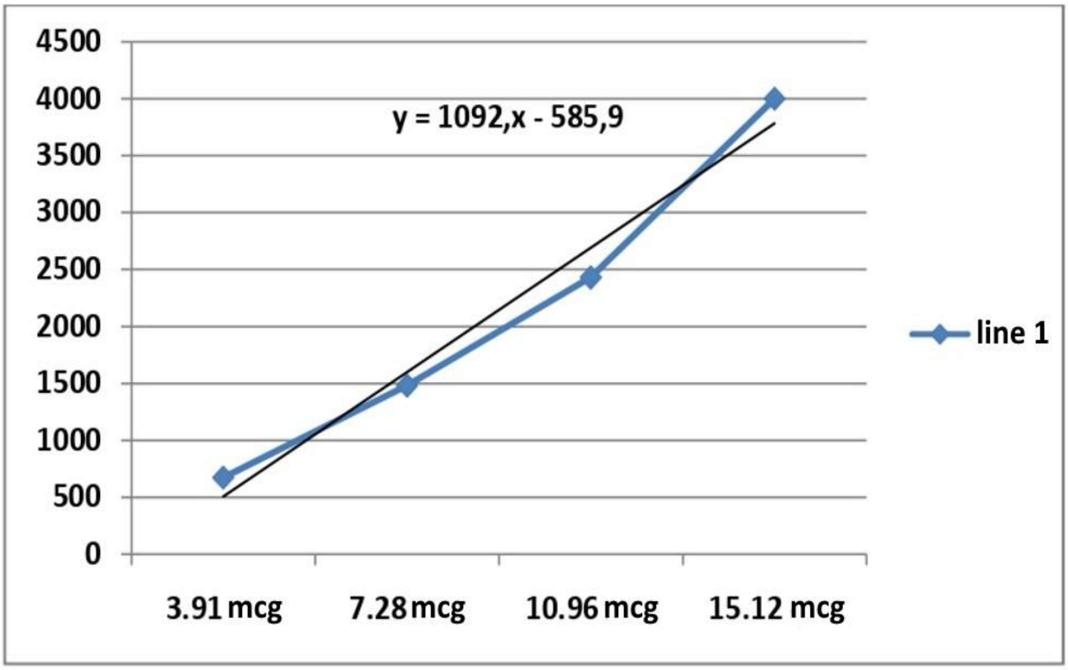

Figure S4. Hydrophobin HPLC calibration.
